# The Importance of Age-Friendly City on Older People’s Continuity and Life Satisfaction

**DOI:** 10.3390/ijerph18147252

**Published:** 2021-07-06

**Authors:** Siew-Imm Ng, Xin-Jean Lim, Hui-Chuan Hsu

**Affiliations:** 1 School of Business and Economics, Universiti Putra Malaysia, Seri Kembangan 43400, Malaysia; imm_ns@upm.edu.my (S.-I.N.); xinjean.lim@xmu.edu.my (X.-J.L.); 2 Research Center of Health Equity, School of Public Health, College of Public Health, Taipei Medical University, Taipei 11031, Taiwan

**Keywords:** age-friendly city, continuity theory, gender difference, life satisfaction, older adults, structural equation model

## Abstract

According to continuity theory, successful aging is promoted when older people are able to continue familiar activities as a way to maintain self-identity. The purpose of this study was to examine the importance of both external resources provided by Taipei city and older adults’ internal resources in internal and external continuity and life satisfaction. The data were from the 2019 Taipei City Senior Citizen Condition Survey acquired through face-to-face interviews. Only the community-based sample without disability was included in the analysis (*n* = 1494). Structural equation modeling was used for the analysis. Both internal and external resources significantly promoted internal continuity (physical activity, Internet use, and lifelong learning) and external continuity (work, social connectedness, and social participation), and the effects of personal resources were larger. External continuity was positively related to life satisfaction. The effects of external resources on continuity and life satisfaction were stronger in older women than in older men. Age-friendly cities may provide support for activity continuity and promote well-being for older people. Policy suggestions are discussed.

## 1. Introduction

Successful adaptation to aging through the continuity of attitudes and activities developed over the course of life is a key determinant of older people’s life satisfaction [1]. However, continuity ability is dependent on both individual factors (personal or internal resources) and society approval or support (external resources) [2]. Besides individuals’ personal resources (education, health, and financial status), the role of external resources provided by age-friendly city initiatives in promoting older people’s continuity and life satisfaction should not be undermined. The direct effect of the city’s age-friendliness on life satisfaction has been examined [3,4,5]. However, there is little research examining the effects of both external and internal resources on life satisfaction. In addition, there may be gender differences in the support of external and internal resources that cause different effects on life satisfaction. In this study, we examine the sequential process of resources (perceived age-friendliness and personal resources) in promoting continuities (external, including work, social connectedness, and social participation; and internal, including physical activity, Internet use, and lifelong learning) and its impact on life satisfaction with gender as a moderating factor in the case of Taipei’s older population.

Taiwan’s life expectancy has improved significantly in the last decade. Given this, understanding older people’s living environments that support their lifestyles and life satisfaction are necessary in developing more appropriate age-friendly programs. We argue that a supported resources–continuities–satisfaction relationship, would provide insights to Taipei city age-friendly authorities to focus their efforts not only on the older cohort but also the middle-aged. The early cultivation of healthy habits (activities that are cognitively and physically stimulating) among the middle-aged group could encourage them to continue these activities when they are old and ease their adaptations into old age. Programs to safeguard or maintain older people’s personal resources such as finances, knowledge, and health should be emphasized.

Two research questions are examined:

1. Does the sequential process of resources–continuities–life satisfaction manifest in older people living in the Taipei community?
2. Do these sequential relationships have different impacts on older women and older men living in the Taipei community?

### 1.1. Theoretical Explanation: Continuity Theory

Atchley [1,6] defines continuity theory as the process in which older adults maintain continuity in the habits, associations, relationships, preferences, activity, attitudes, and behavior that were developed over a lifetime as an adaptation strategy while transitioning to the third age. Their abilities to maintain continuity, which allows them to sustain their self-concept, makes it easier for them to adjust and adapt to the challenges of the aging process, and this determines their life satisfaction [1,2]. That is, older people’s external (social supports or sanctions) and personal resources (past experience, education, network, and knowledge) combine to influence their continuity ability, which then determines their successful adaptation to aging. In other words, continuity theory suggests that older people who continue with the lifestyles they enjoy, such as the socialization, interaction, connections and work that they prefer, are more likely to enjoy higher life satisfaction. According to continuity theory, older people’s basic inner structure can be maintained by continuing familiar ways of living. Using continuity strategies may help older people adapt to changes in old age and achieve positive health or well-being [7,8].

Internal continuity facilitates one’s ability to embrace inner change while maintaining a stable self-concept [9]. Internal resources such as knowledge, ability, health, and finances, with health and financial status being the key factors, have been associated with older people’s well-being in the long run [10]. Deterioration of health or reduction in financial stability may hamper older people’s continuity of external (e.g., work or social participation) and internal activities (e.g., life-long learning). A weak financial situation may force older people to continue work although they do not enjoy. In addition, internal continuity in older people’s social media use positively predicts life satisfaction [11].

External continuity is defined as a “remembered structure of physical and social environments, role relationships, and activities” [1]. External continuity is related to relationship maintenance with close friends, parents, and adult offspring. The maintenance of meaningful activity, valued relationships, and status were motivations to continue social roles [2]. External continuity has been found beneficial to health and wellbeing in empirical studies. Social participation reduces the effects of depression [12]. Continuity in family-related activities (emotional support for adult children and leisure care for grandchildren) and community-related activities (education, clubs, and religion) were positively related to self-rated health [13].

Resources and continuity may appear very similar but they are clearly differentiated by scholars. Resources refer to the older people’s personal ability (knowledge, education, finances, and health-internal resources) and supports from the social environment (friendly community-external resources) [1]. On the other hand, continuities refer to inner psychological continuity as well as outward social behavior continuity [1]. Internal continuity “happens within individuals’ inner thoughts, reflections, learnings, skills, and dispositions” while external continuity is “manifested in activities, relationships, physical and social environment” [1] (p. 1).

Having high personal resources such as being more educated and in better physical and psychological health, and economic satisfaction were found to predict a higher life satisfaction trajectory of Taiwanese older adults as indicated by a 14-year, five-wave panel data [14]. These individual factors provide older adults with different aging experiences.

External resources are sociocultural in nature and vary from society to society [2]. Some societies are relatively flexible and support older people to continue in their long-term social roles such as paid work, or as mentors or community leaders. An age-friendly city aims to support older people in successful aging, and provide external resources that facilitate functioning of older people. Indeed, provision of physical (e.g., natural environment, transport access) and functional (e.g., welfare, senior center, friendly social activities for older adults) environments were reported to enhance older people’s well-being [12]. Similarly, external resources in the aspect of “perceived accessibility of daily travel”, was found to be related to life satisfaction of older people in Northern Europe [15].

Specifically, the key approach used by older people to maintain continuities in their lives, including: (1) maintain daily and weekly routines such as reading the newspaper, going to church, and Wednesday ladies’ home-group; (2) continue to be the same person, still baking, driving, and dancing; (3) continue living like everyone else, watching TV, cleaning, and doing errands [7]. Preferred internal continuity activities (within the individuals) among American older people who participated in a qualitative study comprise intellectual activities (classes, lectures), writing and painting, home maintenance, and meditation [8].

External continuity is related to relationship maintenance with close friends, parents, and adult offspring [1]. One of the convenient but essential ways to maintain external continuity is interacting with familiar people in a familiar environment [7]. Some of the older people’s preferred external continuity activities were volunteering, social activities such as dinners or gatherings, care-giving, church, and travelling [8]. For Taiwanese older people, external continuity in family-related activities (emotional support for adult children and leisure care for grandchildren) and community related activities (education, club, religion) were found positively related to self-rated health [13].

Besides social networks and social participation, an age-friendly city that focuses on the context of providing people with opportunities for quality of life in old age may affect an individual’s behaviors and values regarding well-being [16]. An age-friendly city can be viewed as the context of the external resources in a city that facilitate the functioning of older people and result in life satisfaction [3,5,14].

Both internal and external continuities help individuals focus on and maintain their strengths and minimize the effects of deficits as normal aging occurs. Older people’s efforts to maintain external continuity can also contribute to their internal continuity [7], that is, social interaction experiences (external continuity) bring richer content for reminiscence (internal continuity). The interplay between older adults’ own perceptions and support/pressure from the social environment determines older adults’ inner psychological continuity and the outward continuity of social behavior and circumstances [1]. As a person’s resources and abilities increase, their ability to continue in social roles increases [2]. For example, a person with high socioeconomic status and corresponding resources can maintain previous social roles much more easily than one lacking such resources. Older people with less desirable roles and fewer resources are unlikely to want to continue previous roles into old age.

Based on the theory and the empirical findings, we hypothesize that older people’s abilities to maintain continuities allow them to sustain their self-concept and make it easier for them to adjust and adapt to the challenges of the aging process, which determines their life satisfaction. Furthermore, the interaction between older people’s own efforts/perceptions (personal resources) and support/effort from the social environment (external resources) determine older adults’ continuity abilities. In short, continuity theory implies that a resources–continuity–satisfaction mechanism exists in explaining older people’s life satisfaction.

### 1.2. Predictors of Life Satisfaction

Life satisfaction is defined as one’s self-evaluation of his/her life as a whole [17]. Life satisfaction has been used as a major proxy measure for people’s successful aging [18] since it reflects not only their objective situation but also their subjective evaluation of life. It has been the central theme in gerontological research where various levels of variables (city level, society level, and individual level) were studied to understand how they influence life satisfaction. All studies concluded that besides individual characteristics, both the societies and cities in which the older people live play significant roles in supporting older people’s life satisfaction [4,10,12,19].

The influences of personal factor on life satisfaction were unanimous across countries. In Spain, Lara et al. [20] investigated the happiness of older Spanish adults. They found that psychosocial resources (health status and social support) predicted older adults’ hedonic balance, life satisfaction, and happiness. The findings support the role played by personal resources in determining life satisfaction. In Brazil, internal resources such as knowledge, ability, health and finances have been found to be associated with older Brazilian people’s well-being in the long run [10]. Similarly, in Taiwan, having higher personal resources such as being more educated and better physical and psychological health, social support, and economic satisfaction were found to predict a higher life satisfaction trajectory of older people based on a 14-year, five-wave longitudinal data [14].

Social relationships and social support play important roles in explaining older people’s life satisfaction and have also been widely researched. Empty-nesters who enjoyed higher levels of social support experienced less loneliness and possessed higher life satisfaction [19]. An individual has to invest time in social relationship development in order to enjoy more social support and achieve a better quality of life [11].

Age friendliness with personal resources also explains life satisfaction. In the study of Flores et al. [3], all eight domains of age-friendly city indicators predicted life satisfaction for the 60–74-year-old cohort, but only some of them (outdoor space and buildings, housing, community support and health services) were significantly related to the 75-year-old-and-above cohort. This seems to imply that there are different impacts of a city’s age friendliness on the life satisfaction of different age cohorts and living conditions. They therefore called for research that focuses on contextual factors. A sense of community may mediate age friendliness (social participation and community and health services) to affect life satisfaction [21]. However, the individual’s characteristics may have more substantial effects than the age-friendliness of a city on life satisfaction [4].

Instead of comparing the relative importance of a city’s age-friendliness and individual factors in driving life satisfaction, it is likely that age-friendliness promotes older people’s continuity, which then determines life satisfaction in a sequential process. This mechanism has been highlighted by continuity theory but has not yet been empirically tested. Although the city’s age-friendliness may be universally beneficial to older people, their effects may vary across countries and across individuals, thus warranting separate research attention in testing it in the context of older people’s life in Taipei.

### 1.3. Gender Differences in Resources, Continuity, and Life Satisfaction

Venn, Davidson and Arber [22] criticized continuity theory for being gender-blind as it did not discuss the different challenges men and women faced in later life, which might affect their adaptation process into old age. That is, the resources owned by older men and older women differ, and their preferred activities to continue also differ, resulting in different impacts on resources and continuities in their adaptation to the aging process. That is, the resources–continuities–satisfaction process may be moderated by gender.

There were discrepancies regarding whether older men or older women adapt better to old age. Gender inequalities in pension provision are possible since long-term and continuous employment was dominated by men while women were largely involved in unpaid jobs such as being homemakers. However, women may be more adaptive to old age than men because they can manage housework, possess wider social networks and enjoy wider interests than men [23]. Older men face different challenges in their transition to old age, such as losses in masculine autonomy and power. Older men and older women are also involved in different types of social activities [18,24]. Thus, the external resources may have different effects on older men and women.

### 1.4. Taipei as an Age-Friendly City

Taiwan will soon become a superaged society in 2025, with at least 20 percent of the population aged 65 or older [25]. Taiwan’s life expectancy reached 80.86 years old, with 77.69 years old for men and 84.23 years old for women, in 2019 [26]. According to the World Health Organization’s age-friendly city guidelines [14], Taiwan is one of the countries in the world with the most age-friendly cities [27].

Taipei in particular has participated in the age-friendly city concept since 2012 in an effort to support sustainable development goal 3, which aims to promote healthy lives and well-being for residents of all ages [28]. Taipei’s Department of Social Welfare (DOSW) was tasked with providing welfare services for senior citizens. The DOSW aims to make Taipei an age-friendly city by facilitating “peaceful aging in place” through various elderly services and activities to promote older people’s participation in social activities, promote mutual care and peer support, and aid in successful aging, including community services, welfare resources, care visitations, telephone greetings and inquiries, referral services, catering services, and health promotion [28]. Taipei identified 325 locations for elderly people to have a meal together. A monthly fare subsidy of New Taiwan Dollar 480 (about USD 17.50) was auto credited into older people’s “Senior Easy Card” for them to enjoy public transport. The subsidy amount is sufficient to cover older people’s daily returned trip for a month. Fourteen seniors’ service centers were established in 12 districts in Taipei, one center in each district (except one of the districts has 3 centers) for elderly’s easier access. The center provides educational and recreational programs, including welfare consultation, association activities, and lecture courses.

**Figure 1:**
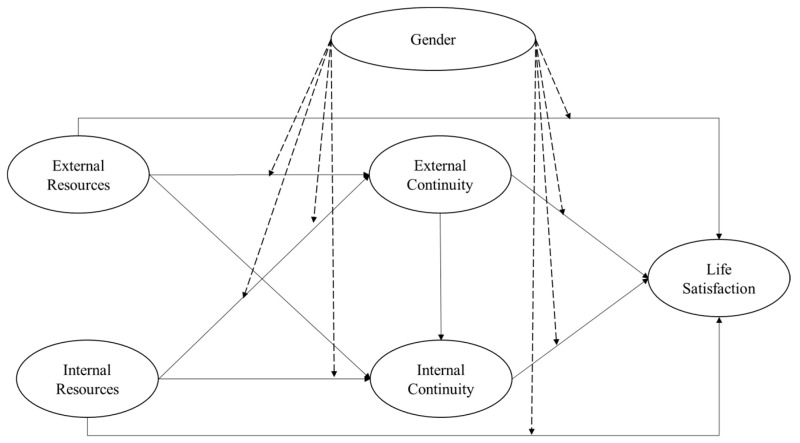
Conceptual Framework Model.

Based on continuity theory and empirical studies, we hypothesized that gender moderates the sequential relationships of resources–continuity–satisfaction, as proposed by continuity theory, as indicated in our proposed framework in Figure 1.

## 2. Materials and Methods

### 2.1. Data and Sample

Our analyses were based on the 2019 Taipei City Senior Citizen Condition Survey [29], conducted by the Department of Social Welfare, Taipei City Government. The sample frame for this survey included people older than 55 years currently living in Taipei city. Multistage sampling was used to proportionally select the respondents. In the first phase, the sample was stratified by age and sex across 12 districts of Taipei city. In the second phase, those samples were selected using systematic sampling according to their address and age. The data were collected by face-to-face interviews. Only the community-based sample without a disability was included in the analysis (*n* = 1494). The data were deidentified when released. The study obtained the approval of the Taipei Medical University Joint Institutional Review Board (No. N202010050).

### 2.2. Measures

Measurement for internal and external resources and internal and external continuities were guided by continuity literature [1]. Internal resources refer to individual’s personal ability, thus measured using three items, including education, financial satisfaction, and self-rated health. External resources are supports from the social environment, in the aspect of perceived age friendliness. In this case, it was measured using two items: “satisfaction with Taipei elderly welfare” and “use of elderly welfare services across a list of ten services provided by Taipei City”. The rationale of using these two items is because high score on satisfaction and broader use experience, indicating the age-friendly services provided are relevant and suited for older people with various needs, thus, demonstrate high friendliness. Internal continuity often happens within individuals in the aspects of inner thoughts, reflections, learnings, skills, and dispositions, thus, this study measured it using three items that reflect on: physical exercise/sport, internet use and lifelong learning. While external continuity is manifested in activities, relationships, and physical and social environment, thus, this was measured using three items that reflect on interaction with family and friends, social interaction via work, and social activities such as volunteering, and working status. Please see the detailed indicators in Table 1.

### 2.3. Analysis

Partial least squares structural equation modeling (PLS-SEM) was utilized in the data analysis to test the sequential process of resources–continuities–satisfaction. PLS-SEM has emerged as a useful analytical tool in the social science disciplines to elucidate how a phenomenon occurs [30]. The proposed relationships were tested using the SmartPLS Version 3.2.3 software, developed by SmartPLS GmbH in Hamburg, Germany. Following the suggested two-stage analytical procedures of the PLS-SEM approach, the measurement model was first evaluated, followed by the structural model [31,32].

## 3. Results

The descriptive analysis of the sample is shown in Table 2. The sample was approximately equally split between genders with 51.90% females and 48.10% males. Most respondents were 65–69 years old (37%), married (78.70%), and had children (96.30%). In terms of living arrangements, 41% were staying in a two-generation family.

The gender differences in the main variables were examined by independent t tests (please see Supplementary Table S1). The analysis gives a microscopic view of how the results might differ based on gender. Overall, the findings showed that older men and older women did not show significant differences in external resources, external continuity, internal continuity, or life satisfaction with the exception of internal resources. In particular, older men showed higher internal resources than older women. The correlation coefficients among the measured variables are reported in Table S2. All main constructs (external continuity, internal continuity, and external resources) were positively correlated to life satisfaction. The quartiles of the variables are shown in Table S3. 

### 3.1. Measurement Model Analysis

**Figure 2:**
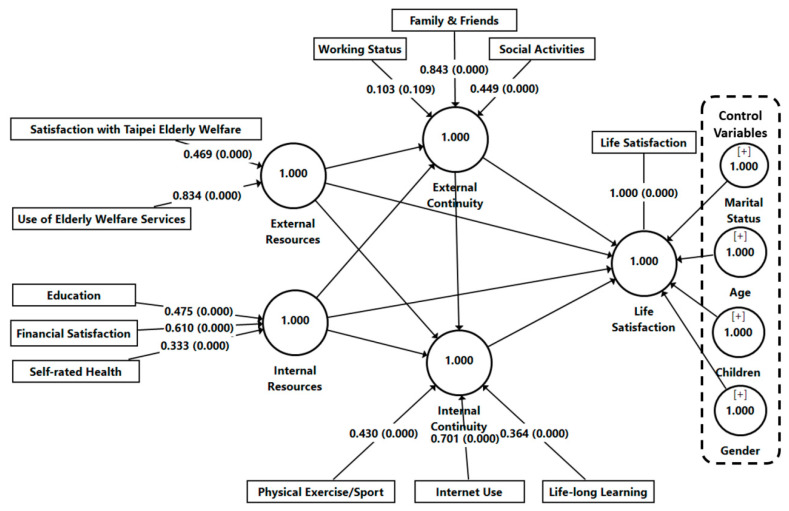
Measurement model.

We examined the measurement model based on the properties of the formative construct, a multicollinearity assessment among items and the significance of the items’ weights [30]. The variance inflation factors (VIFs) were used as the metric to evaluate multicollinearity. As Table 3 shows, multicollinearity issues were nonexistent among all four constructs as the VIF values were below 3.0, ranging from 1.006 to 1.142 [33]. The significance of the formative items was examined using the bootstrapping technique [34]. The findings showed that both items (use of elderly welfare services = 0.834 and satisfaction with Taipei elderly welfare = 0.469) had pronounced significant effects on external resources (*p* < 0.001). Regarding internal resources, three items (financial satisfaction = 0.610, education = 0.475, and self-rated health = 0.333) were statistically significant with *p* < 0.001. Two out of three items of external continuity (social activities = 0.449 and family and friends = 0.843), but not working status, were significant (*p* < 0.001). Nonetheless, this item was retained to fully capture the domains of external continuity, supported by the explanation in continuity theory [1]. Furthermore, internal continuity was found to be significantly (*p* < 0.001) explained by three items, namely, life-long learning (0.364), physical exercise or sport (0.430), and Internet use (0.701) (see Table 3 and Figure 2).

### 3.2. Structural Model Analysis

The structural model was evaluated to assure the model fit [31]. First, the inner VIF was assessed to determine the multicollinearity issue. In our model, the VIFs for all constructs ranged from 1.114 to 1.481 (<3), signifying that multicollinearity is not an issue [33]. Second, the significance of the path relationships was tested via a bootstrapping technique with 5000 subsamples. As for the coefficient of determination (R^2^), external resources, and internal resources accounted for 28.20% and 10.70% of the variance in internal and external continuity, respectively. Approximately 21.4% of the variance in life satisfaction was explained by internal and external resources, as well as by internal and external continuities. Subsequently, the effect size results (ƒ^2^) were interpreted according to Cohen’s (1988) guideline [35], whereby 0.02, 0.15, and 0.35 signify small, medium, and large effect sizes, respectively.

Table 4 details that internal resources (ƒ^2^ = 0.150) were the most important predictor of life satisfaction with a medium effect size while external resources (ƒ^2^ = 0.020) and external (ƒ^2^ = 0.003) and internal (ƒ^2^ = 0.001) continuities had small and trivial effect sizes, respectively. In explaining external continuity, internal resources (ƒ^2^ = 0.089) exhibited a small effect size while external resources (ƒ^2^ = 0.005) exhibited a trivial effect size. Furthermore, when explaining internal continuity, internal resources (ƒ^2^ = 0.100) and external continuity (ƒ^2^ = 0.120) demonstrated small effect sizes while external resources (ƒ^2^ = 0.013) demonstrated a trivial effect size. Finally, the predictive relevance (Q^2^) of the model was analyzed using the blindfolding procedure [36]. Overall, the Q^2^ values for the three endogenous constructs (i.e., life satisfaction, internal continuity, and external continuity) ranged from 0.037 to 0.196 (>0), indicating evidence of the model’s predictive relevance.

**Figure 3:**
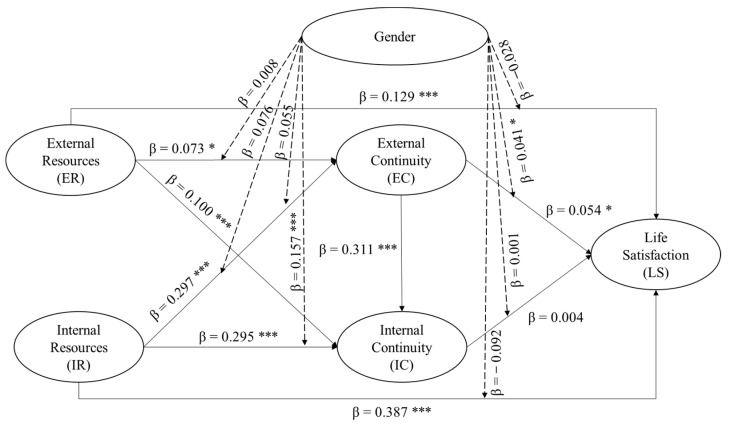
Structural equation model of resources, continuity and life satisfaction for older adults. Note: Indirect Effect: (i) ER → IC → LS: β = 0.001; (ii) ER → EC → LS: β = 0.003; (iii) IR → EC → LS: β = 0.020 *; (iv) IR → IC → LS: β = 0.002.

The results of the estimated parameters of path coefficients are shown in Table 4 and Figure 3. External resources (*β* = 0.129, *t* = 3.780) and internal resources (*β* = 0.387, *t* = 11.213) positively predicted life satisfaction. The results further reported that external resources (*β* = 0.073, *t* = 2.761; *β* = 0.100, *t* = 3.178) and internal resources (*β* = 0.297, *t* = 10.414; *β* = 0.295, *t* = 10.520) positively influence external continuity and internal continuity. External continuity indeed promotes internal continuity (*β* = 0.311, *t* = 12.814) and life satisfaction (*β* = 0.054, *t* = 1.942). However, internal continuity did not significantly influence life satisfaction. We controlled for gender, marital status, age, and children as these variables have been consistently reported to affect life satisfaction [4,20]. In this study, there were no significant effects of marital status, children and gender on life satisfaction. It can be concluded that these factors have negligible impacts on life satisfaction. Nevertheless, a significant relationship was found between age and life satisfaction.

### 3.3. Mediation Analysis

To test the mediating effects, the indirect effects were evaluated based on a bootstrapping approach [37]. As seen in Table 5, only a sequential path (internal resources to external continuity to life satisfaction) was significant, that is, internal resources and life satisfaction were significantly mediated by external continuity (*β* = 0.020, *t* = 2.320), providing partial support to research question 1 on the sequential process of resources–continuities–satisfaction suggested by continuity theory.

### 3.4. Moderation Analysis

The moderating effect of gender was assessed using PLS-SEM to test research question two. A total of eight relationships were tested with gender as a moderator. As presented in Table 6, three relationships were significantly moderated by gender (internal resources and internal continuity, external resources and internal continuity, and external continuity and life satisfaction).

**Figure 4:**
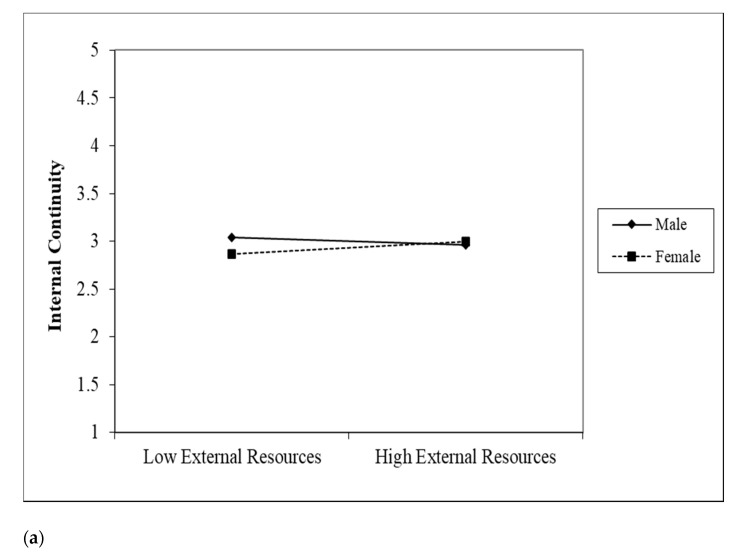

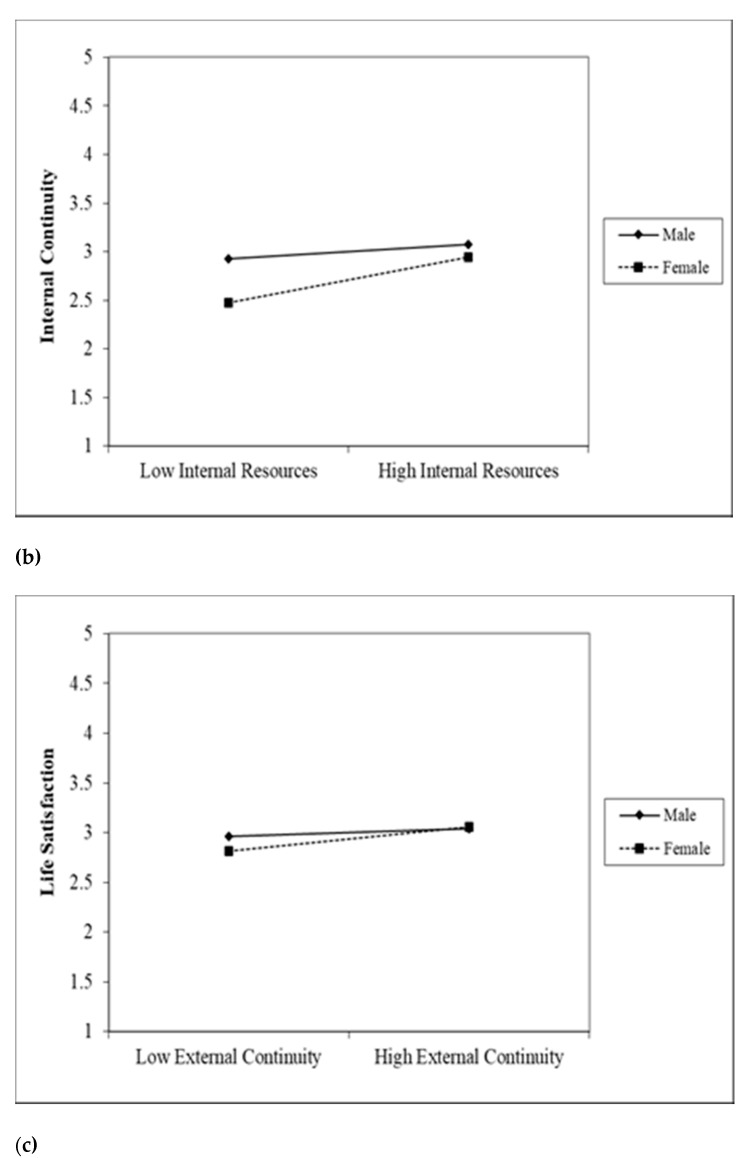
Gender differences in the effects of external and internal resources on continuity and life satisfaction. Note: (**a**) The moderating effect of gender between external resources and internal continuity; (**b**) The moderating effect of gender between internal resources and internal continuity; (**c**) The moderating effect of gender between external continuity and life satisfaction.

These significant relationships were further explained using an interaction plot recommended by Dawson [38]. Figure 4a–c show that the slopes for older women (dotted line) are steeper than those for older men in all three plots, indicating that the positive impact of internal resources and external resources in driving continuities and life satisfaction are stronger for older women than for older men. A closer investigation using model comparison approach (see Table 7) for older men and older women indeed indicated that older women demonstrated higher path coefficients in all three relationships. In fact, external continuity’s influence on life satisfaction was not significant for older men.

## 4. Discussion

This study used structural equation modeling to examine the effects of external/internal resources on life satisfaction through the mediating factors of external/internal continuity and gender as a moderating factor for older people in Taipei. Only one sequential process was found to be significant (internal resources to external continuity and then life satisfaction), providing some support for continuity theory. Most of the proposed direct relationships noted by continuity theory were supported (both internal and external resources determine both internal and external continuities, and external continuity determines life satisfaction). In addition, this study found that the adaptation process to old age differs significantly between older men and older women. In specific, the effects of both internal and external resources in promoting internal continuity, and the effect of external continuity in driving life satisfaction is stronger among older women.

### 4.1. Sequential Process of Resources–Continuity–Satisfaction

To answer the first research question, “Does the sequential process of resources–continuities–life satisfaction manifest in older people living in the Taipei community?”, we tested four sequential processes, but only one significant sequential process was found (internal resources to external continuity and then life satisfaction), providing some support for continuity theory. That is, older people’s internal resources (education, self-rated health, and financial satisfaction) enhance their external continuity (work, social connectedness, and social participation), which determines life satisfaction, an indication of successful adaptation to aging. The relationship in which internal resources predicted life satisfaction is consistent with previous findings [10,14,20]. Older people with greater internal resources are more able to choose and enjoy external activities they prefer, resulting in higher life satisfaction. The internal resources to internal continuity and then life satisfaction link was not significant, probably because internal continuity such as physical activity, Internet use, and lifelong learning does not require financial investment and does not require much physical strength; therefore, it is not limited by internal resources. A deeper investigation found that this is due to the insignificant direct path between internal continuity and life satisfaction (see Table 4). In fact, earlier studies found direct and indirect relationship between internal continuities (internet use and physical exercise) and life satisfaction. Use of social media was found directly and indirectly influenced quality-of-life though perceived social support [1]. Basing on systematic reviews, physical activity was found positively predicted functional mobility, autonomy, anxiety level, balance, social interactions, and life satisfaction [39], suggesting potential mediators (functional mobility, autonomy, anxiety level, balance, social interactions) between physical activity and life satisfaction. That is, internal continuity’s direct influence on life satisfaction is not supported could be due to the presence of mediators. Future studies are needed to clarify these relationships.

External resources (use of elderly welfare services and satisfaction with Taipei elderly welfare) predicted life satisfaction, suggesting that those who used and satisfied with Taipei city’s age-friendly services, tend to enjoy higher life satisfaction. This result was consistent with previous findings [3,4,5]. Surprisingly, the links for external resources to both continuities and then life satisfaction were not supported. In fact, Au and colleagues [21] found age-friendly indicators affect life satisfaction indirectly through sense of community. Mediators used in our studies are activity-related (internal continuities—internet use, life-long learning, and physical activities; and external continuities—working, interaction with family and friends, and participation social activities) and not affective construct. Very likely affective mediators (e.g., sense of communities) exist between continuities and life satisfaction. Future studies are needed to clarify these relationships. Another possibility is related to the measurement of external resources used in this study. One possible explanation is that older adults in Taipei city were not aware of all the welfare services for older people. Another explanation is some older people’s internal and external continuity activities (e.g., watching TV, using the Internet, self-learning, and socializing with family and friends) are less related to age-friendly services.

This result indicates that in the Taipei city context, elderly people’s internal resources are more important than their external resources in helping them maintain their external continuities, which allows them to sustain their self-concept and makes it easier for them to adjust and adapt to the challenges of the aging process, as manifested in life satisfaction [1,2].

### 4.2. Moderating Effect of Gender

There were gender differences in the moderating effects of this model. The effectiveness of internal and external resources in promoting the internal continuity are stronger among older women. External continuity was effective only in driving the life satisfaction of older women and not effective for older men. These findings support the call for using “gendered-lens” in gerontologist research [22]. For example, in the case of widowhood, some older women reported a greater sense of freedom and autonomy, while older men may not view widowhood in the same positive light. Similarly, a gender-sensitive perspective should be considered in age-friendly city policy and services.

### 4.3. Limitations

There are limitations in this study. First, this study used secondary data; thus, only available variables were used in the model. For example, only three indicators each were used to measure internal and external continuity. Second, the measure for life satisfaction used in this study was measured using a single-item measure and not multiple indicators. Although a single global item measure of life satisfaction has been popularly used [3], multiple indicators may more accurately reflect various aspects of life satisfaction. Future studies might want to adopt multiple item measures for life satisfaction. Third, this study did not evaluate the effectiveness of the eight age-friendliness indicators in contributing to the life satisfaction of older Taipei people. Although this is not the aim of the study, evaluating the effectiveness of each indicator may provide insights to cities on which indicators to improve. Future studies might want to evaluate the relative effectiveness of Taipei city age-friendly indicators. Fourth, the study was cross-sectional, thus, causal relationships cannot be confirmed.

## 5. Conclusions

This study examined the relevance of continuity theory’s sequential process (resources–continuities–satisfaction) in explaining older people’s continuities of activities and life satisfaction in Taipei city using gender as a moderator. Internal or personal resources were found to promote continuities and life satisfaction.

This study found evidence for the sequential process of internal resources to external continuities and then life satisfaction, suggested by continuity theory. This indicates that in the Taipei city context, older people’s internal resources are more important than external resources, in helping them maintain their external continuities, allowing them to sustain self-concept, and making it easier for them to adjust and adapt to the challenges of aging process, as manifested in life satisfaction [1,2]. Policy makers could develop programs to safeguard or maintain older people’s resources in terms of health, finances, and education.

We found that Taipei city’s age-friendliness enhances life satisfaction of older people. External resources provided by age-friendly Taipei city did play a role in promoting internal and external continuities and improving life satisfaction. Despite it being agreed that the city’s age-friendliness is generally beneficial to all elderly people, there are some exceptions. We found age-friendly initiatives bring differential benefits to older men and older women. Older men and older women rated external resources very similarly, indicating their usage and satisfaction on age-friendly services were similar. However, these external resources were more effective in promoting an internal continuity for older women. Older women are more likely to be the disadvantaged population due to their lower socioeconomic status and worse health status. An age friendly environment would help to strengthen external resources and then to improve their continuity, which would be beneficial for their well-being. The result also implies that attention should be given by local authorities and voluntary organizations in having dialogs with older men to understand how they wish to be supported. A gender-sensitive approach to design or deliver welfare and age-friendly services is suggested.

By the continuation familiar activities, older people can maintain their perception of self-capability and confidence and help the adaptation process into old age. Taipei city age-friendly programs could be designed with that aim. Ideas for programs can be collected by inviting older people in Taipei for town hall meetings, so that all possible activities familiar to them are recorded and considered. The city can also plan ahead, cultivating a healthier lifestyle and encouraging physical exercise in younger or middle-aged people with the aim that such activities will be continued when they are old.

## Figures and Tables

**Table 1:** Measurement items.

Variable	Item	Scale
External Resources	(i) Satisfaction with Taipei elderly welfare: Are you satisfied with the elderly welfare services currently provided by the Taipei City Government	1 = very dissatisfied to 4 = very satisfied
	(ii) * Use of elderly welfare services: In the past year, have you used the following senior citizen services or concessions provided by the Taipei City Government?” e.g., a fare subsidy of NT480 monthly, concession tickets for cultural centers, utilizing park/walkway/facility space, hiking trails, concession tickets for theatres, seniors service centers, day care services, sport centers, and cultural tours	1 = yes and 0 = no
Internal Resources	(i) Education: What is your highest education level?	1 = illiterate, 2 = self-study/private school/elementary school, 3 = national (junior) middle school, 4 = high school (vocational), 5 = junior college, 6 = university, and 7 = graduate school
	(ii) Financial satisfaction: Are you satisfied with your current financial situation?	1 = very dissatisfied to 5 = very satisfied
	(iii) Self-rated health: How do you feel about your current health?	1 = very bad to 5 = very good
External Continuity	(i) Working status: Are you currently engaged in work?	1 = no and 2 = yes
	(ii) Family and friends: In the past three months, how often did you have social contacts with friends, relatives or colleagues?	1 = never to 7 = everyday
	(iii) * Social activities: In the past year, have you participated in the following activities? Voluntary service, political group activity, religion group activity and other social activities	1 = yes and 0 = no
Internal Continuity	(i) Physical exercise/sport: In the past three months, how often did you engage in physical exercise or sport?	1 = less than once a month to 5 = five times a week or almost every day
	(ii) Internet use: On average, how much time you spend surfing the Internet in the past three months?	0 = never to 4 = everyday
	(iii) Lifelong learning: In the past three months, did you participate in learning activities?	1 = no and 2 = yes
Life Satisfaction	Overall, are you satisfied with your current life?	1 = very dissatisfied to 5 = very satisfied

Note: * Item measures using summated score.

**Table 2:** Characteristics of the sample.

Variables		Mean (SD) or %
Gender	Male	48.10%
	Female	51.90%
Age	60–64 years old	23.00%
	65–69 years old	37.00%
	70–74 years old	19.50%
	75–79 years old	12.70
	80–84 years old	5.20%
	85 years old and above	2.70%
Marital Status	Married	78.70%
	Windowed	14.50%
	Divorced	4.40%
	Single	2.40%
Children	With Children	96.30%
	Without Children	3.70%
Living Arrangement	Live with spouse only	30.70%
	Two-generation family	41.00%
	Three-generation family	25.30%
	Four-generation family	0.30%
	Live with other relatives or friends	2.60%
	Live with caregiver	0.10%
External Resources	Satisfaction with Taipei elderly welfare	2.630 (0.656)
	Use of elderly welfare services	1.718 (1.173)
Internal Resources	Education	3.700 (1.550)
	Financial satisfaction	3.620 (0.757)
	Self-rated health	3.677 (0.775)
External Continuity	Working status	1.150 (0.358)
	Family and friends	5.060 (1.676)
	Social activities	0.423 (0.711)
Internal Continuity	Physical exercise/sports	4.090 (1.266)
	Use of internet	2.550 (1.865)
	Lifelong learning	1.120 (0.322)
Life Satisfaction		3.040 (0.461)

Note: *n* = 1494.

**Table 3:** The parameters of the measurement model.

Formative Variable	Item	Outer VIF	Outer Weight	Standard Error	*t*-Value	*p*-Value
External Resources	(i) Use of elderly welfare services	1.012	0.834	0.055	15.059 ***	<0.001
	(ii) Satisfaction with Taipei elderly welfare	1.012	0.469	0.086	5.425 ***	<0.001
Internal Resources	(i) Financial satisfaction	1.137	0.610	0.049	12.333 ***	<0.001
	(ii) Education	1.051	0.475	0.047	10.062 ***	<0.001
	(iii) Self-rated health	1.142	0.333	0.045	7.340 ***	<0.001
External Continuity	(i) Social activities	1.006	0.449	0.060	7.522 ***	<0.001
	(ii) Family and friends	1.014	0.843	0.037	22.788 ***	<0.001
	(iii) Working status	1.008	0.103	0.060	1.709	0.088
Internal Continuity	(i) Life-long learning	1.045	0.364	0.061	5.926 ***	<0.001
	(ii) Physical exercise or sport	1.022	0.430	0.056	7.632 ***	<0.001
	(iii) Internet use	1.041	0.701	0.045	15.651 ***	<0.001

Note: VIF: variance inflation factor. *** *p* < 0.001.

**Table 4:** The parameters of the structural equation model.

Relationship	Standard Beta	Standard Error	*t*-Value	*p*-Value	VIF	R^2^	F^2^	Q^2^
ER→ LS	0.129	0.034	3.780 ***	<0.001	1.152	0.214	0.020 (S)	0.196
IR → LS	0.387	0.035	11.213 ***	<0.001	1.397		0.150 (M)	
ER→ EC	0.073	0.026	2.761 *	0.003	1.114	0.107	0.005 (T)	0.037
IR → EC	0.297	0.029	10.414 ***	<0.001	1.114		0.089 (S)	
ER→IC	0.100	0.032	3.178 ***	<0.001	1.120	0.282	0.013 (T)	0.111
IR → IC	0.295	0.028	10.520***	<0.001	1.213		0.100 (S)	
EC → IC	0.311	0.024	12.814 ***	<0.001	1.120		0.120 (S)	
EC → LS	0.054	0.028	1.942 *	0.026	1.263		0.003 (T)	
IC → LS	0.004	0.028	0.134	0.443	1.481		0.001 (T)	
**Control Variable**								
Marital Status → LS	−0.027	0.032	0.863	0.194				
Age → LS	0.092	0.024	3.779 ***	<0.001				
Children → LS	−0.038	0.031	1.220	0.111				
Gender → LS	0.028	0.026	1.088	0.138				

Note: LS: life satisfaction; ER: external resources; IR: internal resources; EC: external continuity; IC: internal continuity; LS: life satisfaction. VIF: variance inflation factor; M: Medium effect size; S: Small effect size; T: Trivial effect size, * *p* < 0.05, *** *p* < 0.001.

**Table 5:** Mediating effects of the model.

Mediation Relationship	Indirect Effect	Standard Error	*t*-Values	*p*-Values
ER → IC → Life Satisfaction	0.001	0.004	0.206	0.837
ER → EC → Life Satisfaction	0.003	0.002	1.353	0.177
IR → EC → Life Satisfaction	0.020	0.008	2.320 *	0.021
IR → IC → Life Satisfaction	0.002	0.010	0.218	0.827

Notes: ER: external resources; IR: internal resources; EC: external continuity; IC: internal continuity. * *p* < 0.05.

**Table 6:** Moderating effects of the model.

Moderation Relationship	Standard Beta	Standard Error	*t*-Value	*p*-Value
ER * Gender → EC	0.008	0.028	0.276	0.391
IR * Gender → EC	0.055	0.035	1.557	0.06
ER * Gender → IC	0.098	0.026	3.787 ***	<0.001
IR * Gender → IC	0.157	0.019	8.110 ***	<0.001
ER * Gender → LS	−0.028	0.058	0.487	0.313
IR * Gender → LS	−0.092	0.09	1.026	0.153
EC * Gender → LS	0.041	0.022	1.782 *	0.037
IC * Gender → LS	0.001	0.041	0.013	0.495

**Table 7:** Model comparison for significant moderating relationships by gender.

Relationship	Older Men				Older Women			
	Standard Beta	Standard Error	*t*-Value	*p*-Value	Standard Beta	Standard Error	*t*-Value	*p*-Value
ER → IC	0.130	0.038	3.403 ***	<0.001	0.140	0.049	2.887 ***	<0.001
IR → IC	0.260	0.039	6.623 ***	<0.001	0.312	0.044	7.074 ***	<0.001
EC → LS	0.028	0.04	0.714	0.238	0.087	0.041	2.152 *	0.016

Note: * *p* < 0.05, *** *p* < 0.001.

## Data Availability

We do not have the right to share the data of Taipei City Senior Citizen Condition Survey.

## Supplementary Materials

The following are available online at https://www.mdpi.com/article/10.3390/ijerph18147252/s1, Table S1: Gender differences in external and internal resources, continuity, and life satisfaction, Table S2: Correlations of the variables; Table S3: Quartiles of the variables.
