# Supplementary material for: The Importance of Age-Friendly City on Older People’s Continuity and Life Satisfaction
Source: Int J Environ Res Public Health. 2021 Jul 6;18(14):7252. doi: 10.3390/ijerph18147252 (PMC8306623; doi:10.3390/ijerph18147252)
Supplement: Supplementary file 1 [file ijerph-18-07252-s001.zip › ijerph-1276286-supplementary.pdf]

Table S1. Gender differences in external and internal resources, continuity, and life satisfaction.

Construct	Gender	Mean	Min–Max	SD	*t*-Value	*p* Value
External Resources	Male	2.169	1.00–5.50	0.699	−0.254	0.799
	Female	2.179	1.00–6.00	0.706		
Internal Resources	Male	3.801	1.67–5.67	0.723	7.200	0.000
	Female	3.533	1.33–5.67	0.716		
External Continuity	Male	2.215	0.67–4.00	0.647	−0.009	0.993
	Female	2.215	0.67–3.67	0.643		
Internal Continuity	Male	2.622	0.67–3.67	0.801	1.529	0.126
	Female	2.558	0.67–3.67	0.828		
Life Satisfaction	Male	3.060	1.00–4.00	0.445	1.310	0.191
	Female	3.020	1.00–4.00	0.475		

Note: *n* of male = 718; *n* of females = 776. Analysis by *t* test.

Table S2. Correlation of the variables.

Variables	Age	External Continuity	External Resources	Internal Resources	Internal Continuity	Life Satisfaction
Age	1					
External Continuity	−0.172	1				
External Resources	−0.012	0.167 *	1			
Internal Resources	−0.197	0.32	0.319 ***	1		
Internal Continuity	−0.312	0.422 ***	0.247 ***	0.427 ***	1	
Life Satisfaction	0.009 ***	0.183 *	0.263 *	0.427 ***	0.188	1

Note: Analysis by Pearson's correlation. Age (ordinal). * $p < 0.05$, *** $p < 0.001$.

Table S3. Quartiles of the variables.

Variable	Item	1st Quartile	2nd Quartile (Median)	3rd Quartile
External Resources	(i) Satisfaction with Taipei elderly welfare	2.00	3.00	3.00
	(ii) Use of elderly welfare services	1.00	1.00	2.00
Internal Resources	(ii) Financial satisfaction	3.00	4.00	4.00
	(iii) Self-rated health	3.00	4.00	4.00
External Continuity	(ii) Family & friends	4.00	5.00	7.00
	(iii) Social activities	0.00	0.00	1.00
Internal Continuity	(ii) Internet use	0.00	4.00	4.00
	(iii) Lifelong learning *	1.00	1.00	1.00
Life Satisfaction		3.00	3.00	3.00

Note: * Item measured using dichotomous scale (i.e., 1 = Yes; 0 = No)
